# Orthognathic Surgery to Improve Facial Profile: Assessment, 3-Dimensional Planning, and Technique

**DOI:** 10.1093/asjof/ojaa051

**Published:** 2020-11-19

**Authors:** Kitae E Park, Seija Maniskas, Omar Allam, Navid Pourtaheri, Derek M Steinbacher

**Affiliations:** 1 Section of Plastic and Reconstructive Surgery, Department of Surgery, Yale School of Medicine, New Haven, CT, USA; 2 Yale Division of Plastic and Reconstructive Surgery, New Haven, CT, USA; 3 Plastic and Reconstructive Surgery, Yale School of Medicine, New Haven, CT, USA

## Abstract

A concave profile with class III malocclusion is most often due to a combination of maxillary hypoplasia and mandibular hyperplasia. Surgical correction entails normalization of jaw positions and is more challenging in the setting of concurrent asymmetry and open bite. Treatment should optimize both facial harmony and occlusion. Orthognathic surgery for class III deformities occurs at skeletal maturity and should address all aspects of the condition while preventing unnecessary emotional stress from delayed treatment. In this article, the authors describe the 3-jaw orthognathic surgery technique to address maxillary hypoplasia, mandibular prognathism, open bite, and mandibular asymmetry in a single procedure. The process of preoperative 3-dimensional (3D) virtual surgical planning, detailed surgical technique, fat grafting, and a comparison of preoperative and postoperative 3D aesthetic outcomes is presented. Additionally, a retrospective review of postoperative outcomes of 54 patients who received 3-jaw orthognathic surgery is presented as well.

A concave facial profile is often underpinned by a class III malocclusion and dentoskeletal relationship. Perinasal hollowing, difficulty with lip closure, and an anterior crossbite are frequent characteristics. The condition occurs due to either a retrognathic maxilla, prognathic mandible, or a combination of both. Asymmetric prognathism and an open bite can make the surgical movements more complex.

Orthognathic surgery is a powerful tool to establish an aesthetic facial profile, correct the occlusion, and improve the quality of life in these patients. The surgical movements involve advancing the maxilla, deprojecting the mandible, or some combination of both. Three-dimensional (3D) tools, including virtual surgical planning (VSP) and photogrammetry, are used to precisely determine the amount of advancement and setback. In this article, we demonstrate the use of VSP, fat grafting, and the technical aspects of simultaneous LeFort 1, bilateral sagittal split osteotomy (BSSO), and genioplasty, also known as 3-jaw orthognathic surgery, to correct class III malocclusion, jaw asymmetry, and open bite in a patient with both maxillary hypoplasia and mandibular prognathism. Additionally, we present the postoperative outcomes of 54 patients who underwent 3-jaw surgery at a single institution.

## METHODS

### Indications

A 15-year-old woman presented with a prominent chin, midface deficiency, underbite, and open bite. Mammelons were present on the incisors at the time of examination, suggesting a longstanding open bite. The mandibular midline and chinpoint were deviated to the right, suggestive of asymmetric prognathism. On smiling, the patient had a poor maxillary incisal display with obvious tongue show between the open bite. Orthognathic surgery was planned to correct her maxillary hypoplasia and mandibular prognathism. 

The surgery was planned 3-dimensionally. A digital model of the patient’s bite with teeth in the presurgical position was first taken to use as the basis for final occlusion planning and splint fabrication. Three-dimensional photographs, radiographs, cephalometric measurements, and models are also taken to analyze the patient’s deformities and facial relationships. A webinar-based virtual planning session with the attending surgeon and a technician was then planned 5–6 days before the surgery date. In this session, the maxilla, mandible, and chin were sequentially moved through a variety of planes (eg, sagittal, transverse, yaw, and roll) to achieve a desirable orthognathic relationship. The digital plan was then used to fabricate intermediate and final splints to use intraoperatively. In this patient, it was determined that a combination of Le Fort I advancement, BSSO with setback, yaw correction with rotation, and rotational genioplasty with shortening and advancement would equilibrate facial skeletal position and balance. (See Video 1, which demonstrates a 15-year-old woman with the described condition and procedure.) 

### Operative Technique

BSSO was performed first, followed by Le Fort I osteotomy and genioplasty. Distal portions of the proximal segments were removed in an unequal fashion to restore symmetry to the mandible. The piriform apertures were contoured and opened during Le Fort I to improve breathing. An intermediate splint was used following BSSO to set the mandible position in reference to the uncut maxilla. A second and final splint was used following Le Fort I osteotomy to achieve the final maxilla position in reference to the repositioned mandible. Bone from the resected mandible was harvested and grafted to reconstruct the anterior nasal spine. Fat harvested from the abdomen was processed by Telfa rolling and injected into the face at the end of the procedure.

The patient is admitted postoperatively for observation. A pureed diet is maintained for 3 weeks, followed by a soft diet for another 3 weeks. The patient is seen for follow-up at 1 and 6 weeks from surgery. Three-dimensional images are obtained at each interval up until the past 1-year follow-up. Postoperative images from the video were taken at 4 weeks follow-up.

### Retrospective Chart Review

In addition to the individual patient, an Institutional Review Board-approved retrospective chart review (HIC#1101007932) was performed for consecutive patients who received simultaneous LeFort 1, BSSO, and genioplasty during the period between January 2018 and August 2019. Inclusion criteria included patient undergoing 3-jaw orthognathic surgery with 3-D planning and more than 1 year follow-up. Patients were excluded if they did not meet any of these criteria. Informed consent was obtained for all patients. Patients with no follow-up were excluded from the analysis. Patients were assessed for their postoperative outcomes and complications. Furthermore, patient satisfaction with the outcome was assessed based on their subjective feedback during follow-up visits and cosmetic complaints.

## RESULTS

In total, 55 patients who received 3-jaw surgery were identified during the given time frame. One patient was excluded from analysis due to lack of any follow-up visit. The mean age at the time of surgery was 24.4 years (range: 15-58 years). The mean time of follow-up was 259 days (range: 37-551 days). The mean procedure length was 5.2 hours (range: 3.5-6 hours), and 100% of patients received fat grafting to the face. One patient expressed cosmetic concern following surgery. Three patients returned to the operating room for sterile hardware removal, while two patients experienced temporary postoperative dehydration (Table 1).

**Table 1.** Patient Demographics and Surgical Outcomes

Characteristic	Value
Total patients	54
Age (years)	24.4 (range: 15-58)
Gender	
Male	28 (51.9%)
Female	26 (48.1%)
Mean follow-up (days)	259 (range: 37-551)
Procedure length (min)	312 (range: 229-521)
Length of stay (days)	2.2 (range: 1.3-5.1)
Fat graft	54 (100%)
Complications	
Cosmetic complaint	1 (1.9%)
Hardware removal	5 (9.3%)
Dehydration	5 (9.3%)

## DISCUSSION

In this article, we present the orthognathic surgery for the correction of skeletal class III malocclusion with overbite and jaw asymmetry. Relative to other forms of malocclusion, treatment of class III malocclusion requires additional considerations due to the involvement of both the maxilla/anterior cranial base and mandible. Three-jaw surgery can address this multifactorial condition with a good outcome.

Orthognathic surgery should be performed when longitudinal skeletal growth is close to completion, approximately 2 years after menarche in women.^1^ The presented patient was 15 years old and 2-year postmenarche when she received surgery. In males, the mandible may continue to grow into the early 20s. Intervention was not postponed in this woman, as she had completed growth and we sought improved psychosocial and functional outcomes as soon as feasible.

The patient’s malocclusion was complicated by asymmetry of the mandible and maxillary hypoplasia with narrowing of the nasal airway and breathing difficulty. In addition to the 3-jaw surgery, opening the piriform and reconstructing the anterior nasal spine further address functional issues. Fat grafting can enhance postoperative wound healing, combat inflammation, and promote optimal aesthetic outcomes while minimizing edema.^2^

Retrospective review of patients who underwent 3-jaw surgery revealed a high rate of cosmetic satisfaction, with only 1 patient experiencing dissatisfaction with their final outcome. Patients receiving 3-jaw surgery often present with complicated malocclusive patterns requiring multi-planer movements. As a result, a longer operation time and an increased complication rate can be expected relative to isolated orthognathic procedures.

Three-dimensional planning in orthognathic surgery has unique advantages over conventional planning, allowing the surgeon to perform the assessment, treatment planning, and splint fabrication on a single platform.^3^ The technique is especially useful in cases involving multiple jaws and/or complicating asymmetry. Splint fabrication through the same digital module prevents the risk of cumulative error that can occur when using multiple models as in the case of conventional planning.^3^ Postoperative results of orthognathic surgery planned virtually demonstrate good conformity with presurgical plans and improved aesthetic outcomes compared with conventional planning.^4,5^

Limitations of orthognathic surgery as the main treatment modality to improve facial profile include standard risks associated with surgery and the larger impact on daily life during the recovery phase relative to orthodontic treatment. VSP is associated with a start-up cost and learning curve to effectively use 3D planning software. Nonetheless, the high fidelity of the results and excellent aesthetic outcomes render both modalities attractive options, especially in cases with multiple movements and complicating asymmetry.

## CONCLUSION

Orthognathic surgery is a powerful method to improve the profile. Advances in 3D planning, 3D photography, and adjunctive fat grafting can help enable optimal results.

## References

[1] Xu L, Nicholson P, Wang Q, Alén M, Cheng S. Bone and muscle development during puberty in girls: a seven-year longitudinal study. J Bone Miner Res. 2009;24(10):1693-1698. 19419294 10.1359/jbmr.090405

[2] Cabrejo R, Sawh-Martinez R, Steinbacher DM. Effect of fat grafting on postoperative edema after orthognathic surgery. J Craniofac Surg. 2019;30(3):698-702. 30807474 10.1097/SCS.0000000000005287

[3] Steinbacher DM. 3D analysis, planning, and model surgery. In: Steinbacher DM, eds. Aesthetic Orthognathic Surgery and Rhinoplasty. Hoboken, NJ: Wiley-Blackwell; 2019:53-75.

[4] Wilson A, Gabrick K, Wu R, Madari S, Sawh-Martinez R, Steinbacher D. Conformity of the actual to the planned result in orthognathic surgery. Plast Reconstr Surg. 2019;144(1):89e-97e. 10.1097/PRS.0000000000005744 31246828

[5] Liao YF, Chen YA, Chen YC, Chen YR. Outcomes of conventional versus virtual surgical planning of orthognathic surgery using surgery-first approach for class III asymmetry. Clin Oral Investig. 2020;24(4):1509-1516. 10.1007/s00784-020-03241-4 32100114

